# miRNA profiling shows shared signatures in pediatric asthma, obesity and their comorbidity

**DOI:** 10.3389/fimmu.2026.1792996

**Published:** 2026-05-13

**Authors:** Harshita Shailesh, Mohamed Nadhir Djekidel, Safa Noor, Lena Hayati, Stefan Worgall, Souhaila Al Khodor, Ibrahim Janahi

**Affiliations:** 1 Department of Pediatric Medicine, Division of Pulmonology, Sidra Medicine, Doha, Qatar; 2 Research Department, Sidra Medicine, Doha, Qatar; 3 Department of Pediatrics, Weill Cornel Medical College, New York, NY, United States; 4 Department of Pediatrics, Weill Cornel Medicine-Qatar (WCM-Q), Doha, Qatar

**Keywords:** asthma, biomarkers, children, miRNA profiling, obesity, pediatric

## Abstract

**Rationale:**

Childhood obesity is a known risk factor for asthma and contributes to increased disease severity and reduced corticosteroid responsiveness. However, the molecular mechanisms underlying this comorbidity remain unclear. Using a cross-sectional design, we aimed to identify microRNA (miRNA) signatures associated with asthma–obesity comorbidity in children to shed light on potential shared molecular drivers.

**Methods:**

Whole blood samples were collected from four pediatric groups: normal weight with asthma (NW-A, n = 11), overweight/obesity with asthma (OO-A, n = 10), overweight/obesity without asthma (OO, n = 10), and normal weight without asthma (NW, n = 12). Circulating miRNA profiles were assessed using the NanoString nCounter platform. Differential expression and pathway enrichment analyses were performed using Enrichr and other bioinformatic tools. Correlation with clinical and cytokine data was assessed by Pearson’s correlation and multiple regression analyses.

**Results:**

miRNA expression profiles differed markedly across the four groups. Five miRNAs (miR- 423-3p, -92a-3p, -4536-5p, -197-3p, -891a-5p) were consistently upregulated, and two (miR-144-3p, -641) were downregulated across asthma alone, obesity alone, and asthma-obesity comorbidity. Target gene analysis of OO-A-associated miRNAs highlighted involvement in IL-4, IL-13, and PIP3/AKT signaling, as well as pathways linked to innate immunity and metabolism. In OO-A, miRNA dysregulation was correlated with elevated neutrophils, pro-inflammatory cytokines, and reduced lung function.

**Conclusion:**

In this exploratory study, we identified a shared circulating miRNA signature in asthma–obesity comorbidity. These miRNAs may serve as biomarkers and potential therapeutic targets for stratifying and managing asthma in children with obesity, pending validation in longitudinal studies.

## Graphical Abstract

**Figure:**
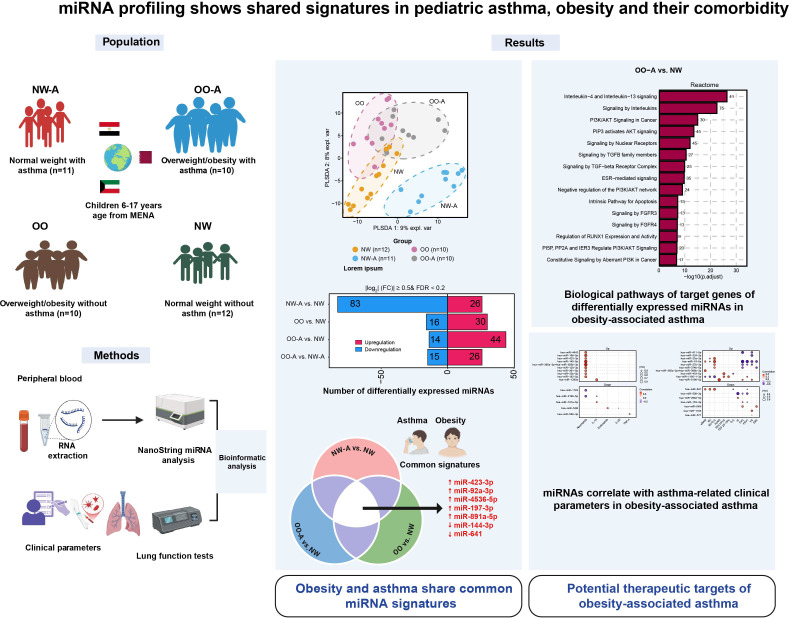
This study investigated miRNA signatures in the peripheral blood of children with obesity and asthma. Total RNA was extracted from whole blood samples of children grouped into four categories: normal weight with asthma (NW-A), overweight/obesity with asthma (OO-A), overweight/obesity without asthma (OO), and normal weight without asthma (NW). miRNA expression was measured using the NanoString nCounter system. Differentially expressed miRNAs were identified through pairwise comparisons between groups, and the miRNAs commonly altered in asthma alone, obesity alone and their comorbidity were identified. Target genes of differentially expressed miRNAs in OO-A were subjected to biological pathway enrichment analysis. Finally, correlation analyses were performed to evaluate associations between differentially expressed miRNAs and clinical parameters, including plasma cytokine levels. Findings: Five miRNAs (miR-423-3p, -92a-3p, -4536-5p, -197-3p, -891a-5p) were consistently upregulated, and two (miR-144-3p, -641) downregulated across asthma, obesity, and comorbidity, indicating shared molecular features. miRNAs associated with asthma-obesity comorbidity were enriched in IL-4, IL-13, and PIP3-AKT signaling pathways. Their dysregulation was also associated with increased inflammation and reduced lung function. MENA, Middle east North Africa; DE, differentially expressed.

## 1 Introduction

Asthma is a heterogeneous inflammatory lung disease that affects approximately 300 million people worldwide and is characterized by airway inflammation and impaired lung function (1, 2). Obesity is a significant contributing factor to childhood asthma. A meta-analysis by Malden et al. reported that obesity increases the incidence of asthma by 50% (3). Moreover, increased adiposity before the age of six is linked to a higher risk of developing childhood asthma (4). These two conditions interact in ways that influence asthma pathobiology (5), as evidenced by more severe symptoms and more frequent exacerbations in obese children compared to their normal-weight peers (6, 7).

The proposed mechanisms underlying this interaction are both immunological and mechanical. Obesity worsens asthma through chronic low-grade inflammation, driven by adipose tissue expansion and the release of pro-inflammatory cytokines such as TNF-α, IL-6, and leptin (8). These cytokines and adipokines promote inflammation by activating Th1 and Th17 immune cells (8, 9). This pro-inflammatory state can amplify immune reactivity to environmental allergens, contributing to airway hyperresponsiveness (8). Mechanically, increased abdominal fat and excess body weight reduce respiratory muscle efficiency, further impairing lung function (10). Despite these observations, the pathophysiological mechanisms linking obesity to asthma severity remain underexplored. Given that children with both comorbidities tend to respond poorly to corticosteroid therapy (6, 7), there is an urgent need to better understand the underlying molecular and immunological pathways. Such insights could support the development of more effective, targeted interventions for this high-risk group.

miRNAs are small (20–22 nucleotide) non-coding single-stranded RNAs that regulate gene expression at transcriptional and post-transcriptional levels (11). They play key roles in the development of inflammatory diseases such as asthma and obesity (12–18). For example, Tiwari et al. (2022) reported a group of blood miRNAs (miR-451b, -7-5p, -532-3p, -296-5p, and -766-3p) associated with exacerbation in pediatric asthma (19). Another study by Zhang et al. (2022) identified 161 differentially expressed miRNAs in allergic asthmatic children, including 21 novel miRNAs (19). Despite these advances, the miRNA signatures associated with asthma–obesity comorbidity in children remain unexplored.

Here, we hypothesized that specific miRNA signatures associated with pediatric asthma–obesity comorbidity could provide insight into the underlying mechanisms and support the development of biomarkers and targeted therapies. To test this, we profiled whole-blood miRNA expression in four groups of children: normal weight with asthma (NW-A), overweight/obesity with asthma (OO-A), overweight/obesity without asthma (OO), and normal weight without asthma (NW). We compared their miRNA profiles, investigated the associated biological pathways, and explored correlations with clinical parameters, including plasma cytokine levels and lung function indices.

## 2 Methods

### 2.1 Study participants

This exploratory study is part of the Sphingolipids in Obesity and Asthma in Pediatrics (SOAP) project—a comprehensive cross-sectional investigation aiming to characterize physiological, genetic, epigenetic, metabolomic, and lipidomic factors influencing asthma and obesity in children residing in Qatar. The study protocol was approved by the institutional review board of Sidra Medicine (IRB No. 1500770). Recruitment of the participants to the study began in August 2017 and continued through December 2023.

For the present analysis, a total of 43 children were selected from the SOAP cohort and assigned to four study groups: NW-A (normal weight with asthma, n = 11), OO-A (overweight/obesity with asthma, n = 10), OO (overweight/obesity without asthma, n = 10), and NW (normal weight without asthma, n = 12) (Graphical abstract). Recruitment procedures and group classification criteria have been described in detail previously (20). The inclusion and exclusion criteria of the study are described in the **Supplementary Material.**

Verbal or written assent was obtained from all participants, along with written consent from parents or legal guardians.

Lung function tests and cytokine assay were performed as described previously (20). Additional details are described in the **Supplementary Material**.

### 2.2 RNA extraction

RNA was extracted from whole blood collected in PAXgene Blood RNA tubes and processed according to the manufacturer’s instructions (see **Supplementary Material** for full protocol).

### 2.3 miRNA expression and downstream analyses

Total RNA was used to profile miRNA expression using the NanoString nCounter Human v3 miRNA Expression Assay (NanoString Technologies, Seattle, WA, USA). The assay targets a panel of 798 preselected miRNAs in addition to 26 control probs. The resulting nCounter dataset was used to analyze differential expression. Downstream analyses including target identification, gene enrichment, biological function analysis, mapping of biological functions to miRNAs, correlation with clinical parameters, and interaction analysis were performed using a range of bioinformatic tools (see **Supplementary Material** for details).

### 2.4 Statistics

Descriptive statistics are reported as means and standard deviations for normally distributed variables, and as medians with interquartile ranges (IQRs) for skewed data. Categorical variables are presented as counts and percentages. Group comparisons were made using one-way ANOVA or the Kruskal–Wallis test for continuous variables, and the chi-square test for categorical variables. Partial correlation analyses between miRNA expression and clinical features were adjusted for patient group. Partial correlation analysis was conducted in an exploratory framework due to small sample size. Significance was defined combining a relatively loose p < 0.1 and stringent |β| > 0.5 to prioritize robust effect size. No formal multiple testing was applied.

Differential expression p-values were estimated using the limma package (v3.58.1), applying relaxed criteria for significance to retain miRNAs large-enough changes to be biologically meaningful. |log_2_(fold change)| ≥ log_2_(1.5) and FDR < 0.2 All statistical analyses were performed using R software (v4.3.2), except for those reported in **Table 1**, which were conducted using STATA (Version SE/17, StataCorp LLC, College Station, TX, USA).

**Table 1:** Basic characteristics of study participants (n=43).

Variables	Normal weight with asthma (NW-A; n = 11)	Overweight/obesity with asthma (OO-A; n = 10)	Overweight/obesity without asthma (OO; n = 10)	Normal weight without asthma (NW; n = 12)	*p* value^†^
Age years^a^	11.05 (3.6)	13.14 (2.8)	13.49 (2.0)	10.5 (3.5)	0.071
Sex, n (%)^a^					
Male	8 (72.7)	9 (90.0)	6 (60.0)	8 (66.7)	0.476
Female	3 (27.3)	1 (10.0)	4 (40.0)	4 (33.3)	
BMI kg/m^2b^	15.4 (14.5, 17.0)	30.7 (27.4, 33.9)	45.3 (40.6, 48.9)	16.4 (15.8, 17.6)	<0.001
BMI-z score^b^	-0.74 (-1.3, -0.04)	2.35 (2.1, 2.4)	2.72 (2.6, 2.9)	-0.25 (-0.6, 0.3)	<0.001
BMI-percentile^b^	23.0 (9.6, 48.5)	99.1 (98.2, 99.3)	99.7 (99.5, 99.8)	39.7 (26.9, 61.1)	<0.001
Medication					
- Inhaled steroids, n (%)^a^	3 (27.3)	6 (60.0)	–	–	–
- Nasal steroids, n (%)^a^	1 (9.1)	4 (40.0)	–	–	–
- Systemic steroids (IV, oral and others)^a^	4 (36.4)	6 (60.0)	–	–	–
Clinical characteristics					
Eczema, n (%)^a^	3 (27.3)	4 (40.0)	1 (10.0)	0 (0.0)	0.079
Allergies, n (%)^a^	4 (36.4)	4 (40.0)	0 (0.0)	0 (0.0)	0.016
Rhinitis, n (%)^a^	4 (36.4)	6 (60.0)	0 (0.0)	0 (0.0)	0.002
Eosinophils (x 10^9^/L)^b^	0.5 (0.3, 0.6)	0.4 (0.3, 0.5)	0.2 (0.1, 0.4)	0.1 (0.1, 0.2)	0.002
Eosinophilia status (≥0.3 × 10^9^/L), n (%)^a^	10 (90.9)	8 (80.0)	4 (44.4)	1 (8.3)	<0.001
Neutrophils (x 10^9^/L)^b^	2.5 (1.6, 3.9)	4.2 (3.6, 5.2)	4.2 (3.9, 6.6)	1.9 (1.1, 3.3)	0.008
Spirometry					
FEV1% predicted^a^	79.36 (6.9)	74.2 (17.1)	96.6 (7.5)	102.1 (14.0)	<0.001
FVC% predicted^a^	94.0 (5.1)	89.6 (12.0)	99.4 (6.9)	101.4 (13.9)	0.048
FEV1/FVC ratio^a^	84.09 (6.1)	81.8 (12.7)	96.7 (5.1)	99.8 (4.8)	<0.001
FEF25-75% predicted^a^	49.5 (12.1)	50.2 (21.9)	88.4 (17.2)	95.7 (18.3)	<0.001
FeNo (ppb)^b^	62.4 (26.5, 176.6)	62.1 (24.7, 150.5)	12.7 (11.3, 20.0)	13.0 (9.4, 22.4)	<0.001
FeNo (≥20 ppb), n (%)	8 (80.0)	9 (90.0)	3 (3.0)	3 (25.0)	0.002
LCI^a^	7.40 (1.7)	7.02 (0.9)	7.28 (0.5)	6.51 (0.2)	0.148

^a^ Mean (standard deviation).

^b^ Median (Interquartile range).

^†^ One-way analysis of variance (ANOVA) or Kruskal-Wallis or chi square test. BMI, body mass index; FEV1, forced expiratory volume in one second; FVC, forced vital capacity; FEF, forced expiratory flow midexpiratory phase; FeNO, fractional exhaled nitric oxide; IV, intravenous; LCI, lung clearance index.

## 3 Results

### 3.1 Study population

This study included 43 children recruited to a separate cross-sectional study on sphingolipids in childhood asthma and obesity (SOAP) (**Table 1**). The mean age was comparable between children of normal weight with or without asthma (11.05 years (NW-A) vs. 10.5 years (NW)), while slightly higher in those with overweight/obesity (13.4 years (OO-A) and 13.5 years (OO)) regardless of asthma status (p = 0.071). Sex distribution was similar across all groups, with a higher proportion of males than females (p = 0.476). As expected, the median BMI percentile was significantly higher in children with overweight/obesity (99.1% (OO-A) and 99.7% (OO)) compared to those of normal weight (23.1% (NW-A), 39.7% (NW), irrespective of asthma status (p < 0.001).

In the NW-A group, the proportions of children using inhaled, nasal, and systemic (oral or intravenous) steroids were 27.3%, 9.1%, and 36.4%, respectively. In the OO-A group, these values were higher at 60%, 40%, and 60%, respectively. As expected, a higher proportion of children with asthma (NW-A and OO-A) had a history of eczema, allergies, and rhinitis compared to those without asthma (NW and OO). Children with asthma also had significantly higher eosinophil counts (0.5 x 10^9^/L (NW-A) and 0.4 x 10^9^/L (OO-A) vs. 0.2 x 10^9^/L (OO), and 0.1 x 10^9^/L (NW)) (p = 0.002) and a higher prevalence of eosinophilia (p < 0.001) regardless of weight status. Conversely, children with overweight/obesity had higher neutrophil counts (4.2 x 10^9^/L (OO-A and OO) than their normal-weight peers (2.5 x 10^9^/L (NW-A) and 1.9 x 10^9^/L (NW)), irrespective of asthma status (p = 0.008).

Lung function parameters, specifically forced expiratory volume in 1 second (FEV_1_) (p < 0.001), forced vital capacity (FVC) (p = 0.048), FEV_1_/FVC ratio (p < 0.001), and forced expiratory flow at 25%–75% of pulmonary volume (FEF25–75%) (p < 0.001), were lower in both asthma groups (NW-A and OO-A) compared to the non-asthma groups. Fractional exhaled nitric oxide (FeNO) levels (p < 0.001) and the proportion of children with FeNO ≥20 ppb (p = 0.002) were also elevated in children with asthma. A similar, though non-significant, trend was observed for lung clearance index (LCI) (p = 0.148) (**Table 1**). Together, these baseline data confirm expected group-level differences in BMI and inflammatory cell profiles, reinforcing the biological relevance of comparing miRNA expression across children with asthma–obesity comorbidity, asthma alone, obesity alone, and healthy controls.

### 3.2 Asthma, obesity, and their comorbidity show distinct miRNA expression compared to controls

**Figure 1:**
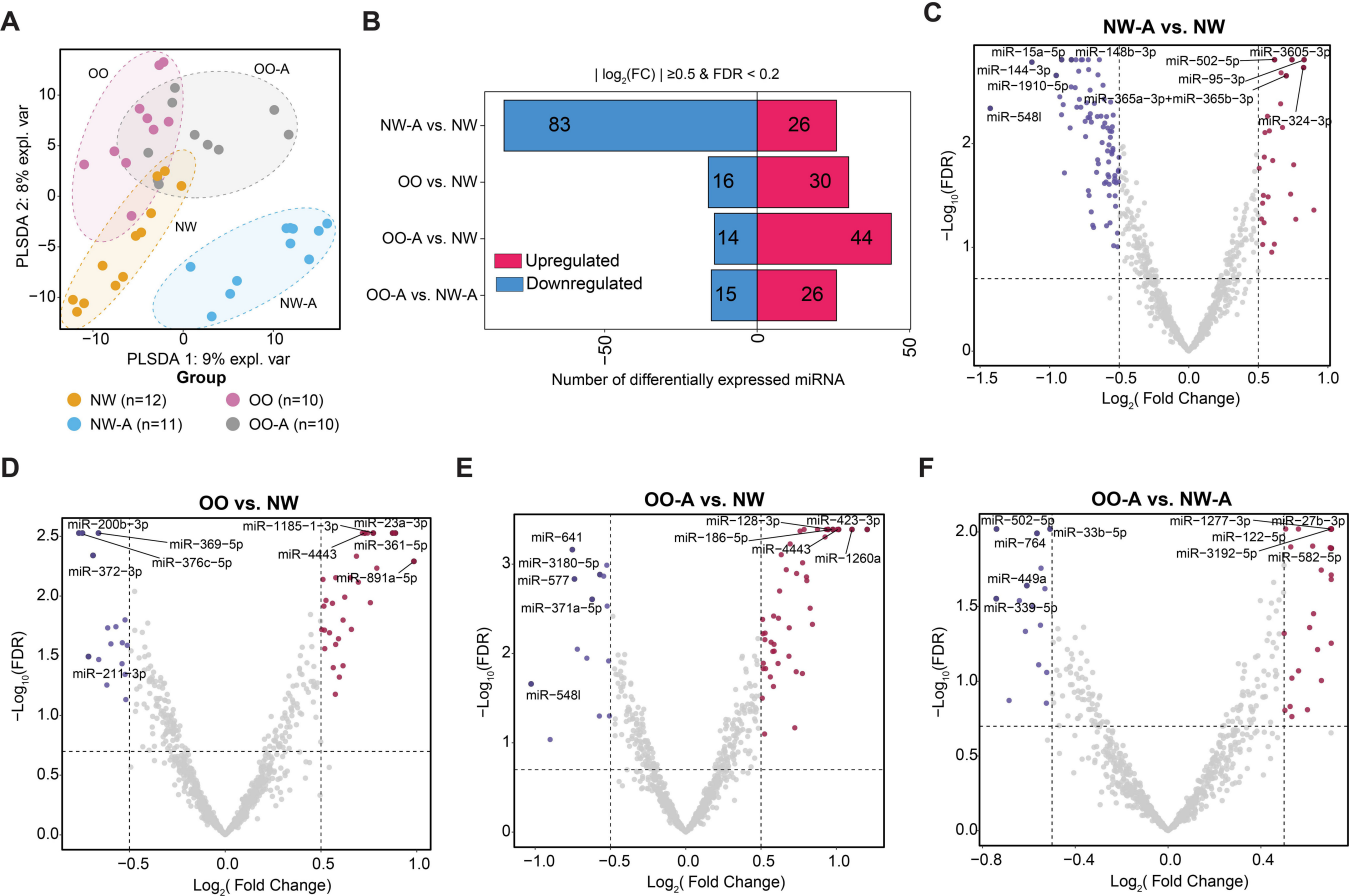
Differential expression analysis of miRNAs across study group comparisons. **(A)** Partial least squares discriminant analysis (PLS-DA) projection based on the most variable miRNAs across four groups: NW-A, OO, OO-A, and NW. Each dot represents a sample, coloured according to group. **(B)** Bar plot showing the number of differentially expressed miRNAs in each group comparison. The x-axis indicates the number of miRNAs; positive values represent upregulated miRNAs (red), and negative values represent downregulated miRNAs (blue). **(C–F)** Volcano plots showing upregulated and downregulated miRNAs in each pairwise comparison: NW-A vs. NW **(C)**, OO vs. NW **(D)**, OO-A vs. NW **(E)**, and OO-A vs. NW-A **(F)**. Significantly upregulated and downregulated miRNAs are shown in red and blue, respectively. The threshold for significance was |log_2_(fold change)| ≥ 0.5 and FDR < 0.2. NW-A, normal weight with asthma; NW, normal weight without asthma; OO, overweight/obese without asthma; OO-A, overweight/obese with asthma.

To evaluate differential miRNA expression across the study groups, we first performed principal component analysis (PCA) using the top 15% of miRNAs with the highest median absolute deviation. The PCA plot revealed varying degrees of separation, with a distinct separation between the NW-A group and the other groups, and more subtle separation among the OO, OO-A, and NW control groups (**Figure 1A**).

We then conducted differential expression analysis to identify specific miRNA signatures that distinguish asthma and obesity phenotypes. Using a significance threshold of FDR < 0.2 and |log_2_(fold change)| ≥ log_2_(1.5), we examined the expression of 798 miRNAs across groups. The comparison between the NW-A and NW groups yielded 109 differentially expressed miRNAs, of which 26 were upregulated and 83 were downregulated (**Figures 1B, C**; **Supplementary Table 1**).

The OO group showed 46 differentially expressed miRNAs compared to the NW group, including 30 upregulated and 16 downregulated miRNAs (**Figures 1B, D**; **Supplementary Table 1**). In the OO-A versus NW comparison, 58 miRNAs were differentially expressed, with 44 upregulated and 14 downregulated (**Figures 1B, E**; **Supplementary Table 1**). Lastly, comparing the OO-A and NW-A groups revealed 41 differentially expressed miRNAs, including 26 upregulated and 15 downregulated (**Figures 1B, F**; **Supplementary Table 1**).

In summary, these findings reveal distinct and overlapping miRNA signatures associated with asthma, obesity, and their comorbidity, providing a foundation for further exploration of shared and condition-specific molecular pathways.

### 3.3 Asthma and obesity are associated with distinct and overlapping miRNA signatures

**Figure 2:**
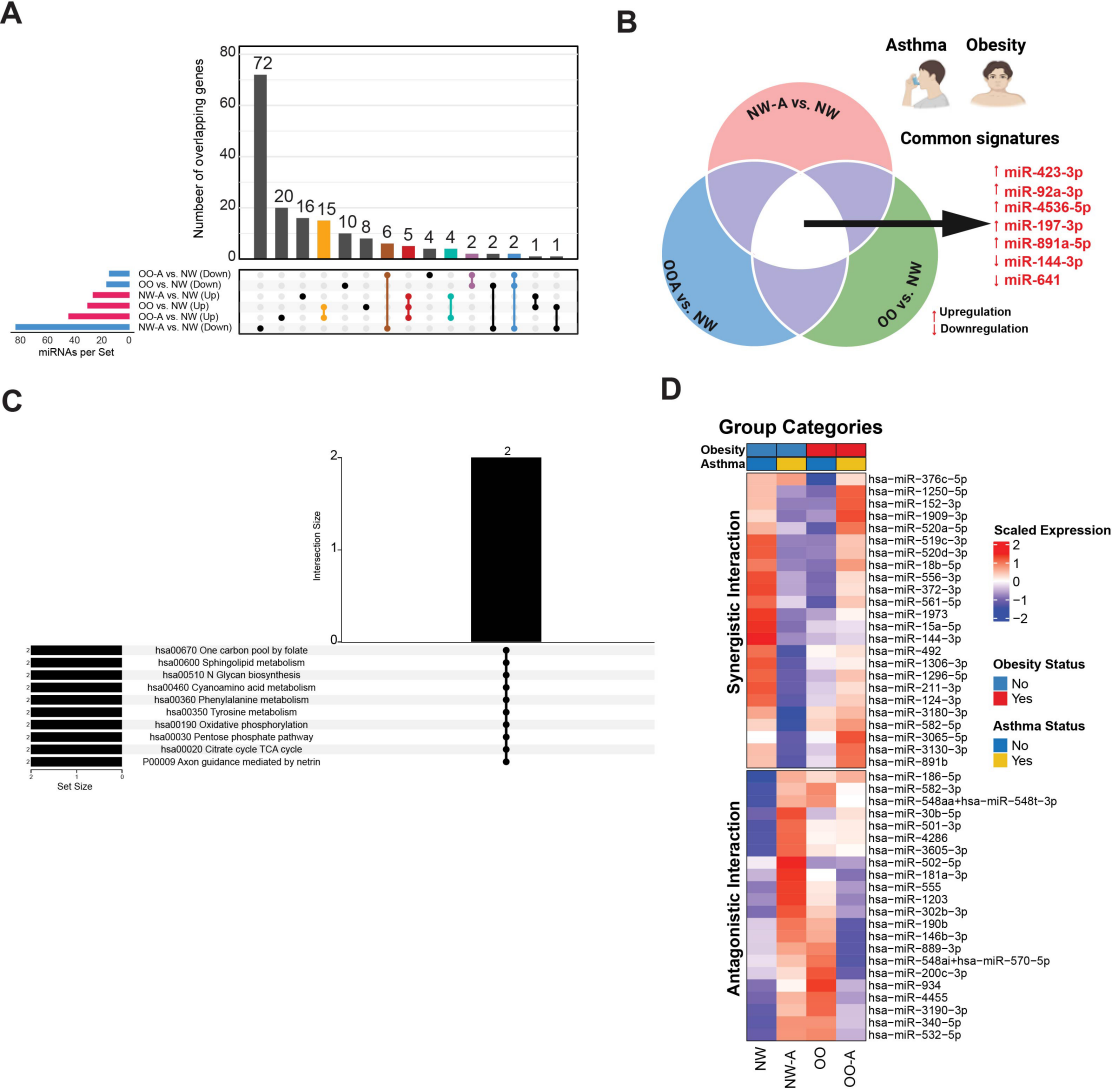
Unique and shared miRNA signatures in asthma, obesity, and comorbidity. **(A)** UpSet diagram showing the number of unique and overlapping upregulated and downregulated miRNAs across different group comparisons. **(B)** miRNAs commonly affected in asthma alone, obesity alone, and asthma–obesity comorbidity. **(C)** Functional enrichment analysis of commonly altered miRNAs in asthma–obesity comorbidity using pathway databases. **(D)** Heatmap showing the scaled expression levels of miRNAs significantly affected by synergistic or antagonistic interaction effects between asthma and obesity. NW-A, normal weight with asthma; NW, normal weight without asthma; OO, overweight/obese without asthma; OO-A, overweight/obese with asthma.

To identify unique and shared molecular features associated with asthma, obesity, and their coexistence, we cross-referenced the differentially expressed miRNAs across these conditions using UpSet plot analysis (**Figure 2A**; **Supplementary Table 2**). We found that 16 miRNAs were uniquely associated with asthma, 9 with obesity, and 20 with the asthma–obesity comorbidity group.

Notably, five miRNAs (miR-423-3p, miR-92a-3p, miR-4536-5p, miR-197-3p, and miR-891a-5p) were consistently upregulated by 0.50 to 1.20 log_2_FC, and two miRNAs (miR-144-3p and miR-641) were consistently downregulated by -0.52 to -1.13 log_2_FC in the NW-A vs. NW, OO vs. NW, and OO-A vs. NW comparisons (**Figure 2B**). Together, these findings highlight distinct and overlapping miRNA signatures associated with asthma, obesity, and their comorbidity, providing a foundation for further exploration of shared and condition-specific molecular pathways.

Functional enrichment analysis of these shared miRNAs using miRWalk revealed significant enrichment in metabolic pathways, including sphingolipid metabolism, glycan biosynthesis, phenylalanine metabolism, and tyrosine metabolism (**Figure 2C**).

Additionally, multiple regression analysis indicated that asthma and obesity interact significantly to alter the expression of several miRNAs (**Figure 2D**). Of these, 24 miRNAs exhibited synergistic interactions and 22 showed antagonistic interactions (**Supplementary Figures 1A, B**). Notably, miR-144-3p, a miRNA downregulated in both asthma and obesity, demonstrated a significant synergistic interaction effect (**Supplementary Figure 1A**). These findings highlight a set of core miRNAs that may serve as shared molecular markers of asthma and obesity, while also revealing distinct regulatory patterns and interaction effects that differentiate comorbid asthma–obesity from either condition alone.

### 3.4 Comparative analysis reveals distinct enrichment of asthma-related biological pathways in asthma–obesity comorbidity

**Figure 3:**
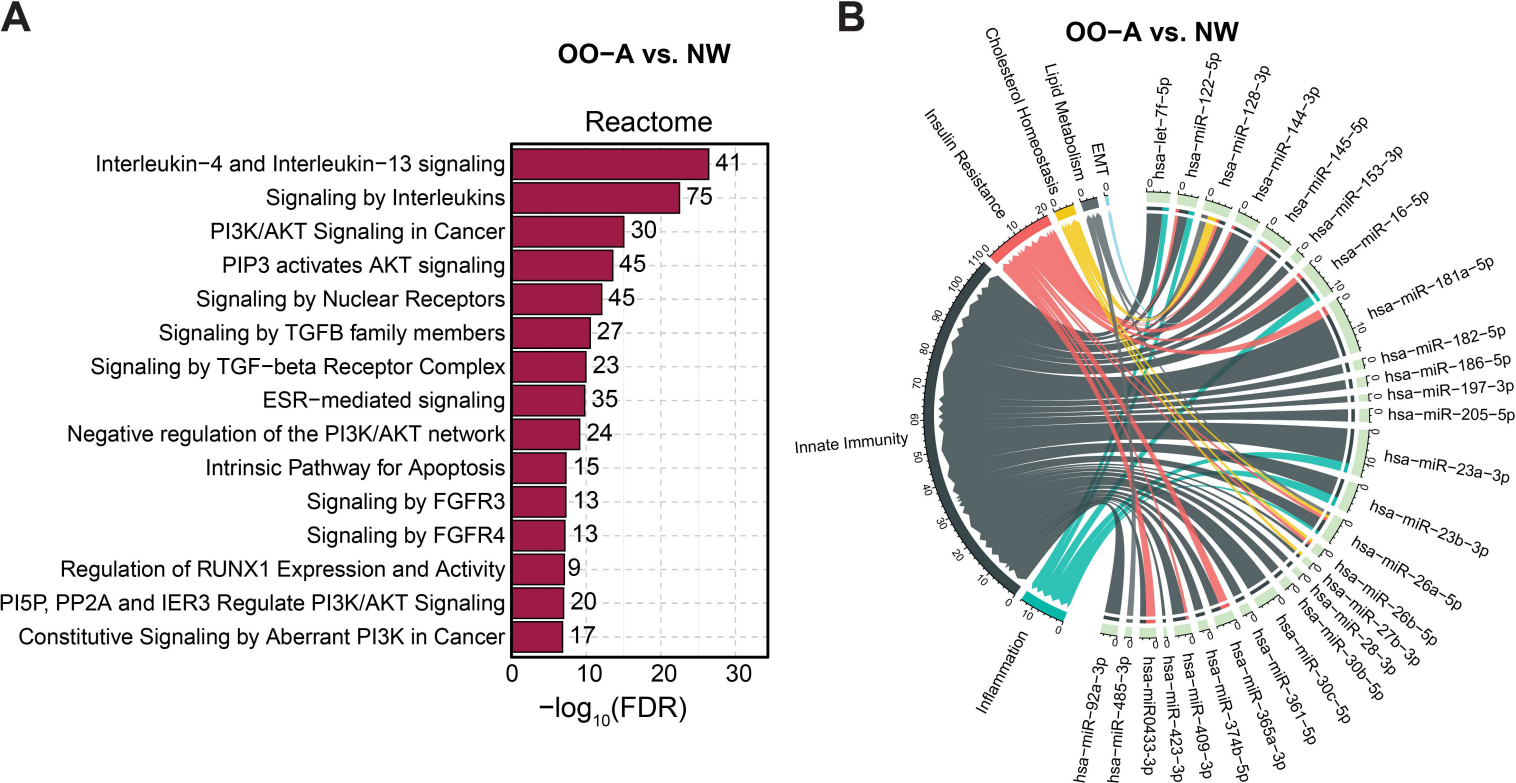
Enrichment analysis of biological pathways associated with miRNAs in asthma–obesity comorbidity. **(A)** Enriched Reactome biological pathways of target genes corresponding to differentially expressed miRNAs in the asthma–obesity comorbidity group compared to the normal-weight healthy control group, identified using Enrichr. The significance range is shown as –log_10_(adjusted p-value), ranging from 2 to 12. **(B)** Circos plot showing the number of miRNA target genes associated with asthma-related biological functions in asthma–obesity comorbidity. NW, normal weight without asthma; OO-A, overweight/obese with asthma.

To explore the potential biological implications of the differentially expressed miRNA profiles observed in asthma–obesity comorbidity, we next examined the target genes and enriched pathways associated with these miRNAs. Target genes of the differentially expressed miRNAs in the asthma–obesity comorbidity group were first identified using miRTarBase (**Supplementary Table 3**) and then subjected to functional enrichment analysis via the Reactome pathways database using the Enrichr platform. Asthma-related pathways regulated by these target genes were ranked by combined score. Among the top enriched pathways were IL-4 and IL-13 signaling, signaling by interleukins, PIP3–AKT signaling, and signaling by nuclear receptors, all of which reached statistical significance (**Figure 3A**).

We further identified miRNAs most strongly associated with biological functions relevant to both obesity and asthma using circos plots (**Figure 3B**). This analysis revealed that innate immunity, insulin resistance, inflammation, cholesterol homeostasis, lipid metabolism, and epithelial-to-mesenchymal transition were the most frequently enriched functional categories in the asthma–obesity comorbidity group. These pathway-level findings provide a mechanistic link between the observed miRNA signatures and key processes implicated in asthma and obesity, supporting the idea that comorbidity may involve coordinated dysregulation of immune and metabolic pathways.

### 3.5 miRNA expression correlates with clinical and inflammatory markers in asthma–obesity comorbidity

**Figure 4:**
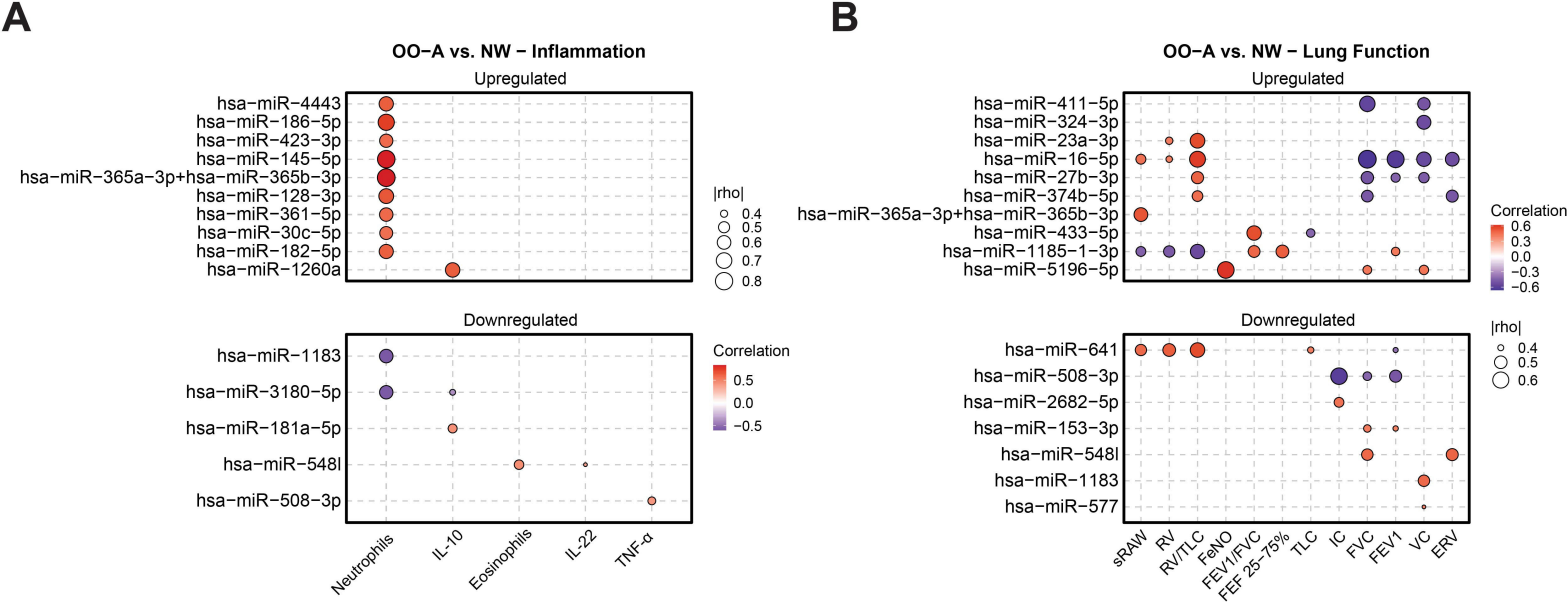
Correlation of differentially expressed miRNAs with clinical parameters and plasma cytokines. Associations between differentially expressed miRNAs and clinical parameters or plasma cytokines in the asthma–obesity comorbidity group were identified using partial correlation analysis, adjusted for patient group. NW, normal weight without asthma; OO-A, overweight/obese with asthma.

We finally explored the clinical relevance of differentially expressed miRNAs in asthma–obesity comorbidity using partial correlation analysis. We found that miRNA expression was associated with clinical indices including eosinophils, neutrophils, lung function parameters, and circulating plasma cytokines, when comparing the asthma–obesity comorbidity group with normal-weight healthy controls (p < 0.1) (**Figures 4A, B**).

Eleven upregulated miRNAs showed positive correlations with inflammatory markers such as neutrophils, IL-10, and FeNO (**Figure 4A**). By contrast, two downregulated miRNAs showed negative correlations with neutrophils and IL-10. Additionally, ten upregulated miRNAs correlated positively with markers of air trapping and resistance (including specific airway resistance (sRAW), residual volume (RV), and RV/total lung capacity (TLC)) and inversely with lung function measures such as FEV_1_, FVC, vital capacity (VC), and ERV (**Figure 4B**). Five downregulated miRNAs showed positive correlations with IC, FEV_1_, FVC, VC, and expiratory reserve volume (ERV), suggesting potential protective associations with lung function. Collectively, these findings highlight specific miRNA signatures in asthma–obesity comorbidity that are not only biologically enriched but also clinically relevant, correlating with markers of airway inflammation, lung function impairment, and systemic immune regulation.

## 4 Discussion

The growing prevalence of both asthma and obesity, and their significant impact on public health, necessitate a deeper understanding of the molecular mechanisms driving these conditions, particularly when they co-occur. miRNAs are key post-transcriptional regulators of gene expression and have been implicated in both immune regulation and metabolic dysfunction, making them strong candidates for investigating the molecular mechanisms underlying diseases such as asthma and obesity (19, 21). However, the role of miRNAs in regulating the pathomechanisms of asthma-obesity comorbidity and as a potential molecular links connecting these two conditions remains largely unexplored. For this reason, we investigated miRNA signatures in the peripheral blood of children with asthma, with and without obesity, to identify miRNAs potentially associated with each condition and their comorbidity. Overall, we found that obesity significantly alters the miRNA landscape in pediatric asthma, with expression patterns that differ from those observed in asthma alone or obesity alone. Notably, five miRNAs (miR-423-3p, -92a-3p, -4536-5p, -197-3p, and -891a-5p) were consistently upregulated, and two miRNAs (miR-144-3p and -641) were consistently downregulated in both the asthma-alone and obesity-alone groups, suggesting shared molecular pathways contributing to the pathophysiology of both conditions. GO enrichment analysis of differentially expressed miRNAs in the asthma–obesity comorbidity group indicated significant enrichment of Th2-related inflammatory pathways. Furthermore, correlation analysis revealed that these dysregulated miRNAs were associated with asthma-related clinical outcomes, suggesting their potential as therapeutic targets.

A recent study by Alhamdan et al. reported differential expression of miRNAs in plasma extracellular vesicles from adults with obese low type 2 asthma and non-obese low type 2 asthma, compared to healthy controls (22). The target genes of differentially expressed miRNAs in obese low type 2 asthma showed enrichment in pathways related to inflammatory cytokine regulation and metabolic processes. Furthermore, the study also identified several clusters of miRNAs in obese low type 2 asthma that correlated with multiple key laboratory and lung function parameters (22). Similar to their study, we also identified distinct miRNA signatures associated with different asthma phenotypes. However, the miRNA signatures identified in our study were different from those identified in the plasma extracellular vesicles by the Alhamdan et al. group. The difference in miRNA signatures may be due to the distinct tissue sources analyzed in our study compared to those used by Alhamdan et al.

Of note, children with asthma alone exhibited the greatest number of differentially expressed miRNAs (n = 109) compared to healthy controls in our study, indicating greater miRNA dysregulation in this phenotype. The obesity-alone group showed fewer changes (n = 46), while the asthma–obesity comorbidity group exhibited 58 differentially expressed miRNAs, including 20 that were uniquely altered in this group. Furthermore, similar to the Alhamdan et al. study, we also found differentially expressed miRNAs linked to inflammatory and metabolic pathways, and correlating with laboratory and lung function parameters in obesity-associated asthma. Importantly, we noted that several miRNAs were consistently upregulated or downregulated across all three groups—asthma alone, obesity alone, and asthma–obesity comorbidity—when compared to the normal-weight control group, indicating a common molecular link between obesity and asthma via miRNAs. However, Alhamdan et al. could not identify such a common molecular link between asthma and obesity, as their study did not include an obesity-alone group.

Among the miRNAs commonly upregulated in our study, miR-423-3p has previously been shown to increase in adipose and lung tissue following allergic sensitization in rats (23). miR-423-3p is involved in the regulation of ERK signaling and has been implicated in cellular processes such as proliferation and migration (24). It also plays a role in lipid metabolism by regulating the expression of apolipoprotein D (25), suggesting a possible metabolic function relevant to both obesity and allergic asthma.

hsa-miR-144-3p has been reported to show elevated expression in both the serum and lungs of adults with asthma, where it correlates with disease severity and corticosteroid use (26). Similar upregulation has also been observed in childhood asthma (27). However, in contrast to these studies, we found that hsa-miR-144-3p was downregulated in the circulation of children with asthma in our cohort. This discrepancy may reflect differences in disease severity, as miR-144-3p expression could vary with asthma severity status. Notably, reduced expression of hsa-miR-144-3p has also been reported in obese women, where it negatively correlated with BMI and serum leptin levels (28). Consistent with this, we observed reduced miR-144-3p expression in children with obesity, further supporting its potential role as a molecular link between the two conditions.

Similarly, reduced serum levels of hsa-miR-15a-5p have been reported in children with bronchial asthma compared to healthy peers (29), and experimental studies have shown that miR-15a-5p can protect mice from developing obesity (30). In line with these findings, we observed reduced expression of hsa-miR-15a-5p in both the asthma and obesity groups in our study, suggesting its potential involvement in the pathogenesis of both conditions.

Our correlation analysis between miRNA expression and clinical biomarkers identified several miRNAs strongly associated with inflammatory markers and pulmonary function. In the OO-A group, upregulated miRNAs showed positive correlations with markers of inflammation, including FeNO, IL-10, and neutrophils, as well as with markers of air trapping such as sRAW and RV/TLC. These same miRNAs showed negative associations with markers of airway obstruction, including FEV_1_ and FVC. These results indicate that differentially expressed miRNAs may have a key role in worsening asthma, potentially by increasing inflammation, airway obstruction, and air trapping, and by reducing lung function in the context of asthma–obesity comorbidity. To further support this, several of the correlated miRNAs in our study have previously been linked to asthma pathogenesis in children, reinforcing their potential as therapeutic targets. For instance, miR-145-5p, which was elevated in the asthma–obesity comorbidity group in our study and associated with neutrophilia, has been shown to be upregulated in the cord blood of children who developed asthma after age 3, as well as in children with active asthma at age 6 (31). Prior studies have also reported increased miR-145-5p expression in the airway epithelium of asthmatic mice, where it promoted epithelial barrier dysfunction and the release of inflammatory cytokines (32). Elevated levels of miR-145-3p have been detected in the exhaled breath condensate of asthmatic children (33), further supporting its potential as a non-invasive biomarker and therapeutic candidate. Conversely, we also observed several miRNAs that exhibited protective associations, correlating with improved lung function and reduced inflammatory markers. Together, these findings underscore the potential utility of miRNAs both as biomarkers and potentially as therapeutic targets in the context of asthma–obesity comorbidity.

### 4.1 Limitations and future directions

The small sample size is the primary limitation of this study; however, participants were carefully selected from a larger cohort to best represent the relevant disease phenotypes. This approach reduced inter-individual variability within subgroups, ensured methodological consistency, and enabled the identification of significant differences between the conditions studied. Moving forward, studies with larger cohorts will be needed to confirm the key observations reported here. Hence, our study was conducted in an exploratory, discovery-oriented framework, in which we focused on selecting miRNA and correlations with moderate to large effect-size rather than a very stringent p-value criteria.

As whole blood was used, hemolysis-linked miRNAs might influence the observed signal. However, inspection of the differential expression pattern of hemolysis associated genes such as miR-92a-3p don’t show a uniform elevation across all contrasts and was only restricted to disease vs control comparisons indicating that it is unlikely to be a result of technical artifacts.

Puberty status is another potential factor that may influence the miRNA profile in the circulation. However, this study did not evaluate the confounding effect of puberty status on miRNA expression as data was not available.

Moreover, while the use of well-defined control groups strengthens the reliability of miRNA profiling, the cross-sectional design of this study limits causal inference. Longitudinal studies, validation in independent cohort, and functional investigations in animal models, will be essential to validate these associations and determine the mechanistic roles of candidate miRNAs. We also plan to integrate other omics datasets, such as transcriptomic or metabolomic data from the SOAP study, to help triangulate the functional relevance of the identified miRNAs. Furthermore, testing the identified miRNA signatures in independent cohorts will also enhance the potential of these miRNAs as candidate biomarkers for asthma–obesity comorbidity. If confirmed, there is potential to develop a stratification tool or diagnostic panel based on these miRNAs to identify high-risk children or distinguish disease endotypes.

## 5 Conclusion

This study highlights the molecular heterogeneity of asthma and obesity in children. By including four distinct phenotypic groups (asthma, obesity, asthma–obesity, and healthy controls) and applying multiple analytical approaches, we identified unique miRNA expression patterns that distinguish asthma, obesity, and their overlap. These findings offer promising avenues for precision medicine, as miRNA profiles may enable more accurate phenotyping, prognosis, and therapeutic stratification in pediatric asthma, particularly when complicated by obesity. The therapeutic potential of specific miRNAs, especially those correlated with worsening asthma clinical parameters, such as miR-145-5p—should be further investigated through both basic and clinical research. In addition, miRNAs shared between asthma and obesity, such as miR-144-3p, warrant further study to evaluate their utility as biomarkers in both conditions.

## Data Availability

The datasets presented in this study can be found in online repositories. The names of the repository/repositories and accession number(s) can be found below: GSE317496 (GEO).
