# Supplementary material for: miRNA profiling shows shared signatures in pediatric asthma, obesity and their comorbidity
Source: Front Immunol. 2026 May 13;17:1792996. doi: 10.3389/fimmu.2026.1792996 (PMC13216767; doi:10.3389/fimmu.2026.1792996)
Supplement: Supplementary file 1 [file DataSheet1.docx]

**A**


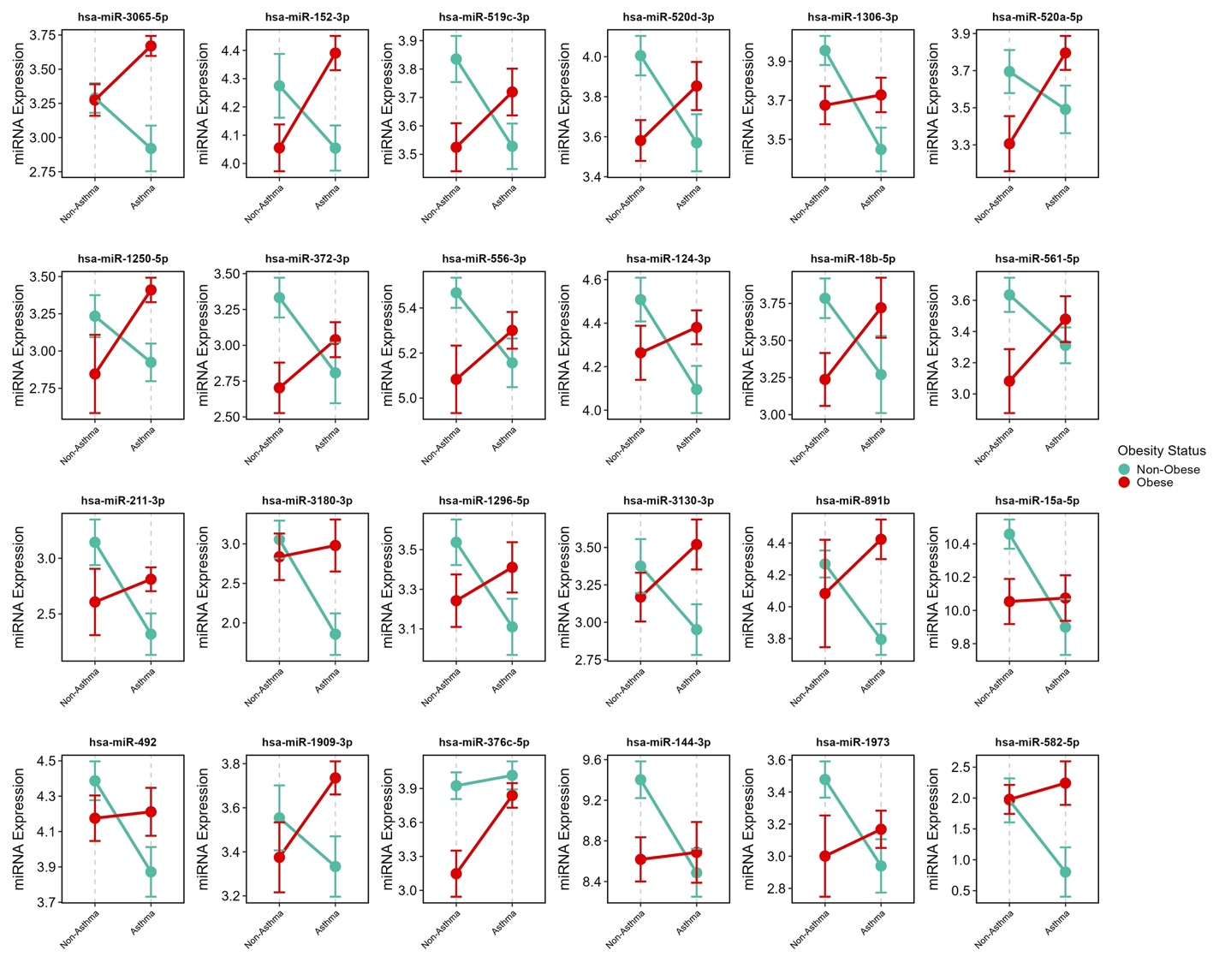


**B**


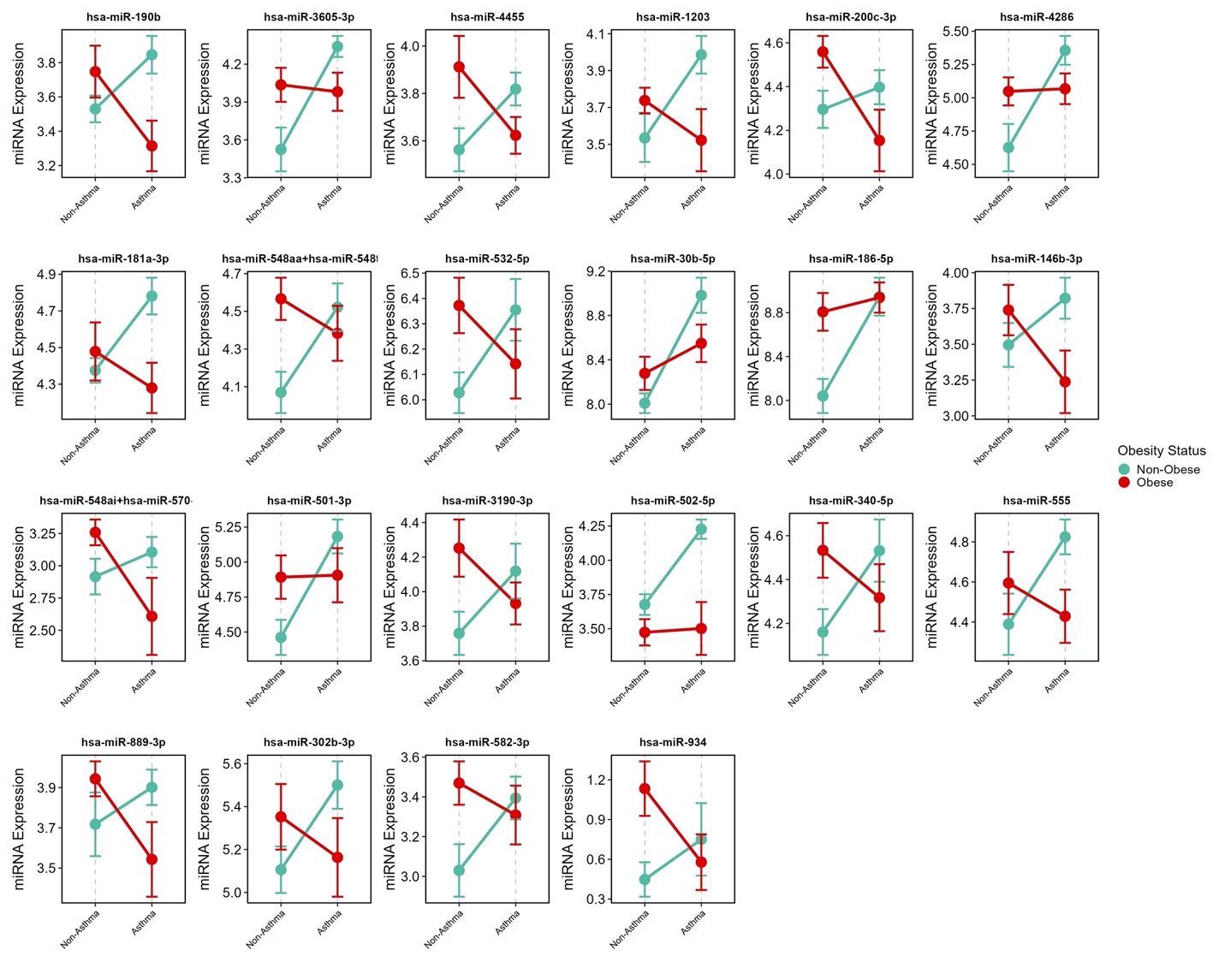


**Supplementary Figure S1:** **Interaction effects of asthma and obesity on miRNA expression.** Multiple regression analysis showing (A) synergistic and (B) antagonistic interaction effects of asthma and obesity on miRNA expression.
