# Supplementary material for: miRNA profiling shows shared signatures in pediatric asthma, obesity and their comorbidity
Source: Front Immunol. 2026 May 13;17:1792996. doi: 10.3389/fimmu.2026.1792996 (PMC13216767; doi:10.3389/fimmu.2026.1792996)
Supplement: Supplementary file 2 [file DataSheet2.docx]

Supplementary Material

# Methods

## Study inclusion and exclusion criteria

Children aged 6 to 17 years residing in Qatar were eligible for inclusion. For the NW-A and OO-A groups, asthma diagnosis was based on physician assessment and a documented history of asthma symptoms, in accordance with the Global Initiative for Asthma (GINA) guidelines (1). A confirmed asthma diagnosis at least 6 months prior to recruitment was required, irrespective of lung function test results. Both, NW-A and OO-A groups, were associated with T2-high asthma phenotype, with either blood eosinophils ≥ 300 cells/µL (0.3 × 10⁹/L) or fractional exhaled nitric oxide (FeNO) ≥ 30 ppb.

Body mass index (BMI) was calculated as weight in kilograms divided by height in meters squared (kg/m²). Overweight and obesity were defined according to age- and sex-adjusted BMI percentiles: ≥85^th^ percentile for overweight and >95^th^ percentile for obesity. Exclusion criteria included the presence of non-asthma chronic lung disease, bronchopulmonary dysplasia, syndromic disorders, inborn errors of metabolism, symptomatic or previously symptomatic congenital heart disease, craniofacial abnormalities, primary thoracic cage deformities, renal impairment, neuromuscular disorders, swallowing difficulties, secondary endocrinopathies causing obesity, or ongoing cancer treatment.

## Lung function tests

Spirometry was performed to measure forced vital capacity (FVC), forced expiratory volume in 1 second (FEV₁), the FEV₁/FVC ratio, forced expiratory flow at 25%–75% of pulmonary volume (FEF 25-75%), peak expiratory flow (PEF), peak inspiratory flow (PIF), and forced expiratory time (FET), as described previously (2).

Body plethysmography was used to assess specific airway resistance (sRAW), vital capacity (VC), inspiratory capacity (IC), functional residual capacity (FRC_pleth_), expiratory reserve volume (ERV), residual volume (RV), total lung capacity (TLC), and the RV/TLC ratio, also as previously described (2). Percent-predicted values for all lung function parameters were used for the analyses.

Fractional exhaled nitric oxide (FeNO) and lung clearance index (LCI) were measured using the Exhalyzer CLD 88 system (Ecomedics, Switzerland) following established protocols (2).

## Cytokine assay

Data on plasma cytokine levels were obtained from our recently published study (3).

## RNA extraction

Whole blood samples (5 ml) were collected from each subject into PAXgene Blood RNA tubes (Cat. No. 762165, BD, USA), incubated at room temperature for 2 hours, and then stored at −80 °C. On the day before RNA extraction, tubes were thawed overnight at 4 °C. Samples were centrifuged at 5000 × *g* for 10 minutes at room temperature, and the resulting pellet was resuspended in 40 μl of RNase-free water. RNA extraction was performed using the PAXgene Blood RNA Kit (Cat. No. 76214, BD, USA), following the manufacturer’s instructions. RNA quality and quantity were assessed by measuring absorbance at 230, 260, and 280 nm using a NanoDrop 8000 spectrophotometer (Thermo Fisher Scientific, Waltham, MA, USA). Only samples with a 260/280 ratio > 1.9 and a 260/230 ratio > 1.8 were included in downstream analyses. Extracted RNA samples were stored at −80 °C until further use.

## miRNA expression analysis

Total RNA stored at - 80°C was thawed on ice. miRNA expression profiling was performed using 100 ng of total RNA on the NanoString nCounter platform (NanoString Technologies, Seattle, WA, USA), targeting 798 preselected miRNAs. Briefly, miRNAs in the RNA pool were multiplex-annealed with specific target tags and ligated. Unligated tags were enzymatically removed following the manufacturer’s protocol (Human v3 miRNA Assay). Tagged samples were processed using the nCounter FLEX system, and digital images were analyzed using the nCounter Digital Analyzer to generate barcode counts in Excel format. Raw counts were normalized using internal controls and reference genes included in the codeset. Quality control (QC) was performed using the nSolver algorithm. Non-human probes were included as negative controls to eliminate background noise.

The resulting nCounter miRNA dataset was analyzed to assess differential expression across study groups. Preprocessing and quality control were performed using the NanoStringNCTools R/Bioconductor package (v.1.10.1) (4). Reporter Code Count (RCC) files were imported using the readNanoStringRccSet function and normalized via the nSolver algorithm. QC assessment confirmed acceptable housekeeping gene expression levels across all samples, and all miRNAs were expressed in more than three individual samples, indicating sufficient detection for downstream analysis. Batch effects were corrected using surrogate variable analysis (SVA), incorporating group and batch as model factors (5).

To explore group-wise differences, partial least squares discriminant analysis (PLSDA) was used to project the data into a latent 2D space (6). Linear modeling was performed using the limma package (7) to identify differentially expressed miRNAs. A relaxed cutoff was applied: |log₂(fold change)| ≥ log₂(1.5) and Benjamini–Hochberg multiple comparison-FDR < 0.2. Results were visualized using volcano plots. Overlap among differentially expressed miRNAs across group comparisons was visualized with an UpSet plot generated using the UpSetR package (v1.4.0) (8).

## Target identification, gene enrichment, and biological function analysis

Target genes and their associated pathways were identified using the miRTarBase database via the MIENTURNET platform (2023-08-09 version), applying a significance threshold of p-value < 0.05 (9). Functional enrichment analysis of these miRNA–target genes was conducted using the Enrichr database (10), incorporating KEGG 2021, Gene Ontology (GO) Biological Processes, and Reactome 2024 pathway datasets, with the default background gene set. Functional terms with a Benjamini–Hochberg adjusted p-value < 0.05 were considered statistically significant.

## Mapping of biological functions to miRNAs

Biological functions relevant to the study were manually grouped into nine functional categories based on shared biological relevance. The number of target miRNAs associated with each category was quantified and visualized using circos plots, generated with the circlize R package (v0.4.16).

## Correlation between miRNA expression and clinical parameters

Partial correlation analysis was performed to evaluate associations between miRNA expression and clinical parameters, including inflammatory markers (eosinophil and neutrophil counts, IL-2, Il-5, IL-6, L-2, IL-5, IL-10, IL-13, IL-17A, IL-22, IL-33, interferon gamma (IFN-γ), tumor necrosis factor alpha(TNF-α), and leptin and lung function parameters (FVC, FEV1, FEV1/FVC, FEF 25-%75%, RV, TLC, RV/TLC, ERV, sRaw, FRCpleth, IC, LCI, and FeNO) was conducted using the prcor.test function from the ppcor R package (v1.1), controlling for patient group. A two-sided p-value < 0.1 was used as the threshold for identifying statistically significant correlations.

## Interaction analysis to identify synergistic and antagonistic effects

To investigate potential interaction effects between obesity and asthma on miRNA expression, linear regression models were fitted for each miRNA using the base R lm() function:

$$expression \sim obese \times asthma$$

miRNAs with a significant interaction term (p < 0.05) and absolute interaction coefficient |β| > 0.5 (to capture more prominent interactions) were considered significantly interacting. Interaction effects were categorized as synergistic (β > 0) or antagonistic (β < 0). Visualizations were generated using the ggplot2 (v3.5.0) (Figure S1) and ComplexHeatmap (v2.18.0) packages (Figure 3D) (11).

**References**

1. Global Initiative for Asthma (GINA). Global Strategy for Asthma Management and Prevention. 2020. (<https://ginasthma.org/wp-content/uploads/2020/06/GINA-2020-report_20_06_04-1-wms.pdf>.).

2. Antonisamy B, Shailesh H, Hani Y, Ahmed LHM, Noor S, Ahmed SY, et al. Sphingolipids in Childhood Asthma and Obesity (SOAP Study): A Protocol of a Cross-Sectional Study. Metabolites. 2023;13(11).

3. Shailesh H, Noor S, Hayati L, Belavendra A, Van Panhuys N, Abou-Samra AB, et al. Asthma and obesity increase inflammatory markers in children. Frontiers in Allergy. 2025;5.

4. Aboyoun P ON. NanoStringNCTools: NanoString nCounter Tools. **R package version 1.16.1** ed2025.

5. Leek JT, Johnson WE, Parker HS, Jaffe AE, Storey JD. The sva package for removing batch effects and other unwanted variation in high-throughput experiments. Bioinformatics. 2012;28(6):882-3.

6. Welham Z, Déjean S, KA LC. Multivariate Analysis with the R Package mixOmics. Methods Mol Biol. 2023;2426:333-59.

7. Ritchie ME, Phipson B, Wu D, Hu Y, Law CW, Shi W, et al. limma powers differential expression analyses for RNA-sequencing and microarray studies. Nucleic Acids Res. 2015;43(7):e47.

8. Conway JR, Lex A, Gehlenborg N. UpSetR: an R package for the visualization of intersecting sets and their properties. Bioinformatics. 2017;33(18):2938-40.

9. Licursi V, Conte F, Fiscon G, Paci P. MIENTURNET: an interactive web tool for microRNA-target enrichment and network-based analysis. BMC Bioinformatics. 2019;20(1):545.

10. Kuleshov MV, Jones MR, Rouillard AD, Fernandez NF, Duan Q, Wang Z, et al. Enrichr: a comprehensive gene set enrichment analysis web server 2016 update. Nucleic Acids Res. 2016;44(W1):W90-7.

11. Gu Z. Complex heatmap visualization. Imeta. 2022;1(3):e43.
