# Supplementary material for: miRNA profiling shows shared signatures in pediatric asthma, obesity and their comorbidity
Source: Front Immunol. 2026 May 13;17:1792996. doi: 10.3389/fimmu.2026.1792996 (PMC13216767; doi:10.3389/fimmu.2026.1792996)
Supplement: Supplementary file 3 [file Table1.docx]

**Supplementary table 1**

List of differentially expressed expressed miRNA with|log₂(fold change)| ≥ log₂(1.5) and Benjamini–Hochberg multiple comparison-adjusted p < 0.2.

| Condition | Upregulated | Downregulated |
| --- | --- | --- |
| NW-A vs. NW | \| hsa-miR-502-5p \| \| --- \| \| hsa-miR-3605-3p \| \| hsa-miR-95-3p \| \| hsa-miR-324-3p \| \| hsa-miR-501-3p \| \| hsa-miR-365a-3p+hsa-miR-365b-3p \| \| hsa-miR-4536-5p \| \| hsa-miR-448 \| \| hsa-miR-4286 \| \| hsa-miR-1228-3p \| \| hsa-miR-3144-5p \| \| hsa-miR-652-5p \| \| hsa-miR-339-5p \| \| hsa-miR-423-3p \| \| hsa-miR-3613-3p \| \| hsa-miR-92a-3p \| \| hsa-miR-532-3p \| \| hsa-miR-874-3p \| \| hsa-miR-4516 \| \| hsa-miR-542-3p \| \| hsa-miR-145-5p \| \| hsa-miR-891a-5p \| \| hsa-miR-197-3p \| \| hsa-miR-548ah-5p \| \| hsa-miR-934 \| \| hsa-miR-656-3p \| | \| hsa-miR-148b-3p \| \| --- \| \| hsa-miR-130a-3p \| \| hsa-miR-15a-5p \| \| hsa-miR-516b-5p \| \| hsa-miR-6503-3p \| \| hsa-miR-491-5p \| \| hsa-miR-3180-5p \| \| hsa-miR-144-3p \| \| hsa-miR-492 \| \| hsa-miR-193b-3p \| \| hsa-miR-760 \| \| hsa-miR-1910-5p \| \| hsa-miR-221-3p \| \| hsa-miR-513b-5p \| \| hsa-miR-378i \| \| hsa-miR-4421 \| \| hsa-miR-1183 \| \| hsa-miR-3690 \| \| hsa-miR-196a-5p \| \| hsa-miR-34c-5p \| \| hsa-miR-330-5p \| \| hsa-miR-526a+hsa-miR-518c-5p+hsa-miR-518d-5p \| \| hsa-miR-211-3p \| \| hsa-miR-506-3p \| \| hsa-miR-514a-5p \| \| hsa-miR-548l \| \| hsa-miR-376a-3p \| \| hsa-miR-1277-3p \| \| hsa-miR-1306-3p \| \| hsa-miR-450a-1-3p \| \| hsa-miR-3074-3p \| \| hsa-miR-124-3p \| \| hsa-miR-1299 \| \| hsa-miR-1285-3p \| \| hsa-miR-660-5p \| \| hsa-miR-323b-3p \| \| hsa-miR-127-3p \| \| hsa-miR-32-5p \| \| hsa-miR-576-5p \| \| hsa-miR-22-3p \| \| hsa-miR-1244 \| \| hsa-miR-3192-5p \| \| hsa-miR-613 \| \| hsa-miR-222-3p \| \| hsa-miR-1264 \| \| hsa-miR-3613-5p \| \| hsa-miR-596 \| \| hsa-miR-153-3p \| \| hsa-miR-378b \| \| hsa-miR-345-5p \| \| hsa-miR-1248 \| \| hsa-miR-491-3p \| \| hsa-miR-556-3p \| \| hsa-miR-873-5p \| \| hsa-miR-568 \| \| hsa-miR-29b-3p \| \| hsa-miR-383-5p \| \| hsa-miR-511-5p \| \| hsa-miR-451a \| \| hsa-miR-7-5p \| \| hsa-miR-15b-5p \| \| hsa-miR-509-5p \| \| hsa-miR-1297 \| \| hsa-miR-142-5p \| \| hsa-miR-1973 \| \| hsa-miR-924 \| \| hsa-miR-539-3p \| \| hsa-miR-548c-5p+hsa-miR-548o-5p+hsa-miR-548am-5p \| \| hsa-miR-1271-3p \| \| hsa-miR-942-3p \| \| hsa-miR-25-3p \| \| hsa-miR-190a-5p \| \| hsa-miR-2110 \| \| hsa-miR-548j-3p \| \| hsa-miR-424-5p \| \| hsa-miR-142-3p \| \| hsa-miR-520a-3p \| \| hsa-miR-641 \| \| hsa-miR-181a-5p \| \| hsa-miR-3180-3p \| \| hsa-miR-300 \| \| hsa-miR-517b-3p \| \| hsa-miR-208b-5p \| |
| OO vs. NW | \| hsa-miR-1185-1-3p \| \| --- \| \| hsa-miR-23a-3p \| \| hsa-miR-361-5p \| \| hsa-miR-4443 \| \| hsa-miR-1260a \| \| hsa-miR-4454+hsa-miR-7975 \| \| hsa-miR-1301-3p \| \| hsa-miR-891a-5p \| \| hsa-miR-128-3p \| \| hsa-miR-362-5p \| \| hsa-miR-129-5p \| \| hsa-miR-665 \| \| hsa-miR-186-5p \| \| hsa-miR-542-5p \| \| hsa-miR-411-5p \| \| hsa-miR-423-3p \| \| hsa-miR-197-3p \| \| hsa-miR-194-5p \| \| hsa-miR-125a-5p \| \| hsa-miR-625-5p \| \| hsa-miR-4536-5p \| \| hsa-miR-25-3p \| \| hsa-miR-934 \| \| hsa-miR-26a-5p \| \| hsa-miR-92a-3p \| \| hsa-miR-1286 \| \| hsa-miR-630 \| \| hsa-miR-5196-5p \| \| hsa-miR-122-5p \| \| hsa-miR-551a \| | \| hsa-miR-200b-3p \| \| --- \| \| hsa-miR-376c-5p \| \| hsa-miR-369-5p \| \| hsa-miR-372-3p \| \| hsa-miR-30a-3p \| \| hsa-miR-18b-5p \| \| hsa-miR-1973 \| \| hsa-miR-577 \| \| hsa-miR-144-3p \| \| hsa-miR-615-3p \| \| hsa-miR-211-3p \| \| hsa-miR-338-5p \| \| hsa-miR-329-5p \| \| hsa-miR-541-3p \| \| hsa-miR-2682-5p \| \| hsa-miR-641 \| |
| OO-A vs. NW | \| hsa-miR-1260a \| \| --- \| \| hsa-miR-423-3p \| \| hsa-miR-128-3p \| \| hsa-miR-26b-5p \| \| hsa-miR-361-5p \| \| hsa-miR-186-5p \| \| hsa-miR-4443 \| \| hsa-miR-92a-3p \| \| hsa-miR-16-5p \| \| hsa-miR-4536-5p \| \| hsa-miR-197-3p \| \| hsa-miR-28-3p \| \| hsa-miR-665 \| \| hsa-miR-27b-3p \| \| hsa-let-7f-5p \| \| hsa-miR-4454+hsa-miR-7975 \| \| hsa-miR-365a-3p+hsa-miR-365b-3p \| \| hsa-miR-26a-5p \| \| hsa-miR-378h \| \| hsa-miR-5196-5p \| \| hsa-miR-23a-3p \| \| hsa-miR-30b-5p \| \| hsa-miR-411-5p \| \| hsa-miR-122-5p \| \| hsa-miR-485-3p \| \| hsa-miR-205-5p \| \| hsa-miR-1185-1-3p \| \| hsa-miR-579-3p \| \| hsa-miR-1301-3p \| \| hsa-miR-30c-5p \| \| hsa-miR-331-3p \| \| hsa-miR-532-3p \| \| hsa-miR-182-5p \| \| hsa-miR-30e-3p \| \| hsa-miR-433-3p \| \| hsa-miR-23b-3p \| \| hsa-miR-374b-5p \| \| hsa-miR-409-3p \| \| hsa-miR-145-5p \| \| hsa-miR-625-5p \| \| hsa-miR-324-3p \| \| hsa-miR-433-5p \| \| hsa-miR-548h-5p \| \| hsa-miR-891a-5p \| | \| hsa-miR-641 \| \| --- \| \| hsa-miR-153-3p \| \| hsa-miR-3180-5p \| \| hsa-miR-1271-3p \| \| hsa-miR-577 \| \| hsa-miR-371a-5p \| \| hsa-miR-181a-5p \| \| hsa-miR-508-3p \| \| hsa-miR-2682-5p \| \| hsa-miR-1183 \| \| hsa-miR-548l \| \| hsa-miR-1972 \| \| hsa-miR-144-3p \| \| hsa-miR-525-3p \| |
| OO-A vs. NW-A | \| hsa-miR-122-5p \| \| --- \| \| hsa-miR-3192-5p \| \| hsa-miR-3065-5p \| \| hsa-miR-1277-3p \| \| hsa-miR-27b-3p \| \| hsa-miR-628-3p \| \| hsa-miR-509-3p \| \| hsa-miR-514b-3p \| \| hsa-miR-1250-5p \| \| hsa-miR-211-3p \| \| hsa-miR-582-5p \| \| hsa-miR-127-3p \| \| hsa-miR-542-5p \| \| hsa-miR-4443 \| \| hsa-miR-23a-3p \| \| hsa-miR-3130-3p \| \| hsa-miR-129-5p \| \| hsa-miR-3180-3p \| \| hsa-miR-125a-5p \| \| hsa-miR-323a-3p \| \| hsa-miR-520e \| \| hsa-miR-433-3p \| \| hsa-miR-671-5p \| \| hsa-miR-548ad-3p \| \| hsa-miR-22-3p \| \| hsa-miR-18b-5p \| | \| hsa-miR-33b-5p \| \| --- \| \| hsa-miR-502-5p \| \| hsa-miR-764 \| \| hsa-miR-95-3p \| \| hsa-miR-449a \| \| hsa-miR-1287-5p \| \| hsa-miR-339-5p \| \| hsa-miR-5010-3p \| \| hsa-miR-652-5p \| \| hsa-miR-152-5p \| \| hsa-miR-548b-3p \| \| hsa-miR-147b \| \| hsa-miR-146b-3p \| \| hsa-miR-604 \| \| hsa-miR-296-5p \| |

Abbreviations: NW-A, normal weight with asthma; NW, normal weight without asthma; OO, overweight/obese without asthma; OO-A, overweight/obese with asthma
