# Supplementary material for: miRNA profiling shows shared signatures in pediatric asthma, obesity and their comorbidity
Source: Front Immunol. 2026 May 13;17:1792996. doi: 10.3389/fimmu.2026.1792996 (PMC13216767; doi:10.3389/fimmu.2026.1792996)
Supplement: Supplementary file 4 [file Table2.docx]

**Supplementary table 2**

List of p-value significant differentially expressed miRNA that are uniquely associated with asthma, obesity, and obesity-associated asthma

| Condition | miRNA |
| --- | --- |
| Asthma only | hsa-miR-502-5p  hsa-miR-3605-3p  hsa-miR-95-3p  hsa-miR-501-3p  hsa-miR-448  hsa-miR-4286  hsa-miR-1228-3p  hsa-miR-3144-5p  hsa-miR-652-5p  hsa-miR-339-5p  hsa-miR-3613-3p  hsa-miR-874-3p  hsa-miR-4516  hsa-miR-542-3p  hsa-miR-548ah-5p  hsa-miR-656-3p |
| Obesity only | hsa-miR-362-5p  hsa-miR-129-5p  hsa-miR-542-5p  hsa-miR-194-5p  hsa-miR-125a-5p  hsa-miR-25-3p  hsa-miR-1286  hsa-miR-630  hsa-miR-551a |
| Obesity- associated asthma only | hsa-miR-26b-5p  hsa-miR-16-5p  hsa-miR-28-3p  hsa-miR-27b-3p  hsa-let-7f-5p  hsa-miR-378h  hsa-miR-30b-5p  hsa-miR-485-3p  hsa-miR-205-5p  hsa-miR-579-3p  hsa-miR-30c-5p  hsa-miR-331-3p  hsa-miR-182-5p  hsa-miR-30e-3p  hsa-miR-433-3p  hsa-miR-23b-3p  hsa-miR-374b-5p  hsa-miR-409-3p  hsa-miR-433-5p  hsa-miR-548h-5p |
